# Rapid antimicrobial susceptibility testing on positive blood cultures through an innovative light scattering technology: performances and turnaround time evaluation

**DOI:** 10.1186/s12879-019-4623-x

**Published:** 2019-11-21

**Authors:** Lidvine Boland, Corentin Streel, Hélène De Wolf, Hector Rodriguez, Alexia Verroken

**Affiliations:** Department of Laboratory Medicine, Microbiology Laboratory, Saint-Luc University Hospital and Catholic University of Louvain, Avenue Hippocrate 10, 1200 Brussels, Belgium

**Keywords:** Positive blood culture, Antimicrobial susceptibility testing, Direct AST, Alfred 60^AST^, Microbiological performances, Turnaround time, Bacteremia

## Abstract

**Background:**

A bacteremia diagnosis with speeded-up identification and antimicrobial susceptibility testing (AST) is mandatory to adjust empirical broad-spectrum antibiotherapy and avoid the emergence of multi-resistant bacteria. Alfred 60^AST^ (Alifax, Polverara, PD, Italy) is an innovative automated system based on light scattering measurements allowing direct AST from positive blood cultures with rapid results. In this study we aimed to evaluate the system’s performances and turnaround time (TAT) compared to routine AST.

**Methods:**

The study was conducted during 2 non-consecutive 3-month periods at the microbiology laboratory of the Cliniques universitaires Saint-Luc. All blood cultures detected positive in the 0 AM–10 AM time frame with a pure Gram-positive cocci or Gram-negative bacilli stain were included for Alfred 60^AST^ testing. Two customized EUCAST antibiotic panels were set up composed of 1) a “Gram-negative” panel including cefuroxime, ceftazidime Enterobacteriaceae, piperacillin-tazobactam Enterobacteriaceae, ciprofloxacine, and ceftazidime *Pseudomonas* 2) a “Gram-positive” panel including cefoxitin *Staphylococcus aureus*, cefoxitin coagulase-negative (CNS) Staphylococci and ampicillin Enterococci. Categorical agreement (CA), very major errors (VME), major errors (ME), minor errors (mE) and TAT to Alfred 60^AST^ results were calculated in comparison with AST results obtained from direct testing on positive blood cultures with the Phoenix system (Becton Dickinson, Franklin Lakes, NJ, USA).

**Results:**

Five hundred seventy and one hundred nine antibiotics were evaluated on respectively 166 Gram-negative bacilli and 109 Gram-positive cocci included in the studied population. During the first study period regarding Gram-negative strains a CA of 89.5% was obtained with a high rate of VME (19 and 15.4% respectively) for cefuroxime and piperacillin-tazobactam Enterobacteriaceae. Considering this, Alifax reviewed these antibiotics’ formulations improving Gram-negative bacilli total CA to 92.2% with no VME during the second study period. For Gram-positive cocci, total CA was 88.1% with 2.3% VME, 13.8% ME (mainly cefoxitin CNS) and 12% mE rates both study periods combined. Median TAT to AST results was 5 h with Alfred versus 12 h34 with Phoenix.

**Conclusion:**

The Alfred 60^AST^ system shows correct yet improvable microbiological performances and a major TAT reduction compared to direct automated AST testing. Clinical studies measuring the impact of the approach on antibiotic management of patients with bacteremia are recommended.

## Background

Bacteremia is characterized by the abnormal presence of bacteria in the bloodstream and can lead to severe complications for the patient as sepsis. This life-threatening condition causes severe organ injuries due to the body’s immune response to the infection [1]. Sepsis furthermore increases morbidity and mortality rates with long-term hospitalizations mainly in intensive care unit (ICU) [2] and the urgent instauration of a broad-spectrum antimicrobial treatment is mandatory to guarantee the patient’s best outcome [3, 4]. However, rapid tailoring of the empirical antibiotherapy with a targeted antimicrobial therapy is subsequently recommended as it can avoid the emergence of multi-resistant bacteria, a major worldwide public health problem of the twenty-first century [5].

Identification and antimicrobial susceptibility testing (AST) of the causative agent has to be considered as an important matter of bacteremia management. Until recently, identification and AST were performed from the subculture of a positive blood culture bottle requiring a turnaround time (TAT) to results of 24–48 h [6]. This delay is now reduced through new genotypic and phenotypic technologies performing the tests directly on the blood of the positive blood culture bottle. For example direct identification with matrix-assisted laser desorption time-of-flight mass spectrometry (MALDI-TOF MS) has been extensively validated with successful identification performances beyond 80% [7, 8]. Complementary molecular-based methods also entered the market offering fast identification combined to the detection of specific resistance genes and requiring a very limited hands-on time yet requiring costly reagents [9]. Considering speeded-up AST testing, many clinical laboratories have now implemented phenotypic AST by direct inoculation using automated platforms enabling results the day after positivity detection [10]. Systems enabling AST results on the day of positive blood culture are just very recently available and stand out with their innovative analysis approaches either based on morphokinetic analysis or on light scattering measurements of the bacteria in the presence of the tested antibiotics [11, 12].

In this study we evaluated the latter approach on the CE-marked automated Alfred 60^AST^ instrument (Alifax, Polverara, PD, Italy). We tested customized panels of antibiotics on all routine positive-detected blood cultures over a 6-month period in comparison with direct automated AST testing. This study aimed at demonstrating acceptable microbiological performances with the Alfred 60^AST^ approach and a reduced TAT compared to routine testing.

## Methods

The study was conducted at the microbiology laboratory of the Cliniques universitaires Saint-Luc – UCL (CUSL), a 979-bed tertiary hospital in Brussels, Belgium. On average 35.000 blood culture pairs are sampled annually with a 3.7% positivity rate. Blood specimens from patients with a suspected bloodstream infection are inoculated into blood culture bottles (BD Bactec Plus Aerobic and Lytic Anaerobic medium, Becton Dickinson, Franklin Lanes, NJ, USA) and incubated 24 h a day, 7 days a week in a Bactec FX device (BD Diagnostic Systems, Sparks, MD, USA). Standard management of positive blood cultures is performed during laboratory working hours (i.e 8 AM – 0 AM, 7 days per week) and includes immediate Gram stain, MALDI-TOF MS identification and automated (Phoenix, Becton Dickinson, Franklin Lakes, NJ, USA) AST.

**Fig. 1:**
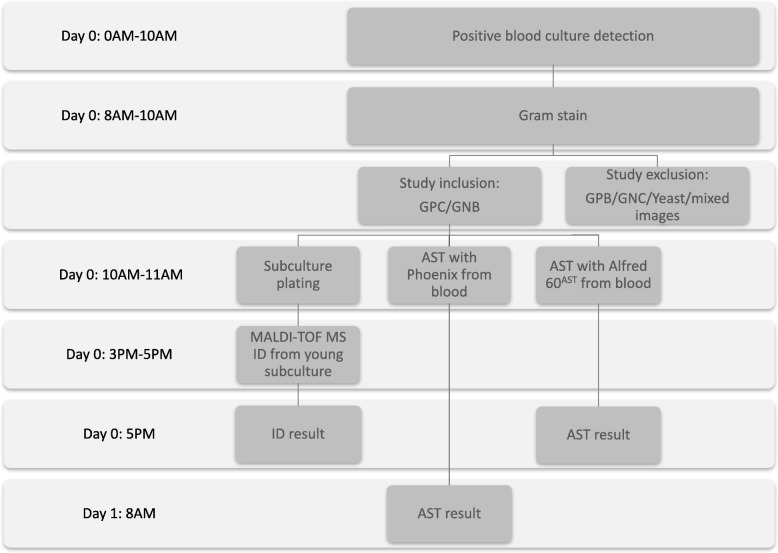
Study inclusion workflow of all blood culture bottles detected positive between 0 AM and 10 AM. AST: antimicrobial susceptibility testing, GPB: Gram-positive bacilli, GPC: Gram-positive cocci, GNB: Gram-negative bacilli, GNC: Gram-negative cocci, ID: identification, MALDI-TOF MS: matrix-assisted laser desorption ionization time-of-flight mass spectrometry

During 2 non-consecutive 3-month periods (February to April 2018 and October to December 2018) all blood cultures detected positive in the 0 AM -10 AM time frame were considered for study inclusion as described in Fig. 1. Gram stain results allowed the inclusion of positive blood cultures with the single presence of Gram-positive cocci or Gram-negative bacilli. Only the first bottle of each positive blood culture episode was used for testing. Each included blood culture was then processed 1) for plating and a 5-h incubation followed by MALDI-TOF MS identification from the young subculture 2) for automated AST directly from the blood with Phoenix and 3) for automated AST directly from the blood with Alfred 60^AST^.

### Direct AST of bacteria using Phoenix

The automated Phoenix system was routinely used at the CUSL microbiology laboratory to assess antimicrobial susceptibility. AST was performed directly from blood of the positive blood culture. Briefly, an 8 ml aspirate of blood from a positive bottle was injected in a Serum Separator Tube (BD Diagnostic Systems, Sparks, MD, USA) and centrifuged at 2000 rpm for 10 min. After removing the supernatant, bacteria on the surface of the gel were suspended into a Phoenix system ID broth tube and put on the BD Phoenix Autoprep instrument to reach a 0.5 McFarland. Ultimately the suspension was inoculated into the appropriate Phoenix panel (NMIC-408 (product no. 448877, BD) for Gram-negative bacilli, PMIC-90 (product no. 448439, BD) for Gram-positive cocci) and incubated in the Phoenix system following the manufacturers’ recommendations.

### AST with Alfred 60^AST^

Antimicrobials tested with the Alfred 60^AST^ approach were chosen from the CE-approved Alifax antibiotic reagent list after discussion with the hospital antimicrobial stewardship team and in concordance with our local resistance epidemiology. Ultimately we established a “Gram-negative bacilli” and a “Gram-positive cocci” panel comprising a selection of EUCAST lyophilized antibiotics. All antibiotics of the selected panel were tested for each included positive blood culture. However upon identification (same-day MALDI-TOF MS testing on young subculture) availability, result performance analysis was exclusively done on the antibiotics interpretable with EUCAST for the identified strain as presented in Table 1. The manufacturer modified the composition of the cefuroxime and piperacillin-tazobactam Enterobacteriaceae (EB) reagents between the 2 study periods.

**Table 1:** Selected antibiotics for result performance analysis following MALDI-TOF MS identification result

		MALDI-TOF MS identification result				
Customized panels	Tested antibiotics	Enterobacteriaceae	*Pseudomonas* *aeruginosa*	*Staphylococcus* *aureus*	Coagulase-negative Staphylococci	Enterococci
Gram-negative bacilli	cefuroxime/EU^a^	X				
	ceftazidime EB/EU	X				
	piperacillin-tazobactam EB/EU	X				
	ceftazidime *Pseudomonas*/EU		X			
	ciprofloxacin/EU	X	X			
Gram-positive cocci	cefoxitin *S. aureus*/EU			X		
	cefoxitin CNS/EU				X	
	ampicillin Enterococci/EU					X

*CNS* coagulase-negative Staphylococci, *EB* Enterobacteriaceae, *EU* EUCAST lyophilized antibiotics, *MALDI-TOF MS* matrix-assisted laser desorption ionization time-of-flight mass spectrometry

^a^Cefuroxime results were not taken into account for AmpC-producing Enterobacteriaceae

Briefly, 10 μl blood of a positive bottle was transferred into a 3 ml enrichment broth vial (Alifax) and loaded in the 37 °C area of the Alfred 60^AST^ instrument with a hands-on time of approximately 15 min for 5 samples. First bacterial growth was monitored through turbidity readings every 5 min and once the suspension had reached a 0.4–0.6 McFarland, it was transferred to an empty vial in the refrigerated zone. Then, 100 μl of the bacterial suspension and 200 μl of each antibiotic from the selected panel were loaded into vials containing a 2 ml enrichment broth (Alifax) for AST analysis in the 37 °C area. One vial containing exclusively the bacterial suspension was used as reference vial. The Alfred 60^AST^ system translated light scattering measurements over time of all vials into growth curves and compared with curves from the reference vial. When resistant bacteria continued to grow, turbidity and light scattering increased; conversely, turbidity remained low and light scattering was reduced when bacteria were susceptible to the tested antibiotic.

### TAT

TAT measurements started when the sample was loaded on Alfred 60^AST^ or on the Phoenix system and stopped when all AST results were made available by the respective system.

Total TAT to results with Alfred 60^AST^ added up a varying TAT to reach a 0.4–0.6 McFarland suspension and a fixed TAT to AST results requiring a 3 h-analysis for all evaluated antibiotics with the exception of cefuroxime, piperacillin-tazobactam Enterobacteriaceae and ceftazidime *Pseudomonas* requiring a 5-h analysis.

### Data analysis

Direct Phoenix testing performed from blood of the positive blood cultures was considered as the reference method to evaluate the microbiological performances of the Alfred 60^AST^ instrument with the selected antibiotic reagents applying the EUCAST 6.0 breakpoints (2016). In case of discrepancy, results were verified by disk diffusion using filter paper disks (Bio-Rad, Marnes-la-Coquette, France) and by minimal inhibition concentration measures using E-test (bioMérieux, Marcy l’Étoile, France) from subcultured colonies. Discordances with cefoxitin antibiotics were verified with an in-house PCR for the detection of the *mecA* gene [13]. Molecular testing was performed on all third-generation cephalosporin-resistant Enterobacteriaceae for the detection of extended-spectrum β-lactamases (ESBL) and of carbapenemases [14].

Alfred 60^AST^ verifications were performed in accordance with the Cumitech recommendations for the verification and validation of procedures in the clinical microbiology laboratory [15]. AST result comparison between Alfred 60^AST^ and the reference method was expressed in a categorical agreement percentage (CA) (total categorical matches / total antibiotics tested × 100). Discordances were classified into very major errors (VME: false susceptibility with the evaluated test), major errors (ME: false resistance with the evaluated test) and minor errors (mE: reference test result intermediate and evaluated test sensitive or resistant, or vice versa).

The VME rate was calculated by dividing the number of VME by the number of resistant bacteria (reference method) × 100. The ME rate was calculated by dividing the number of ME by the number of susceptible bacteria (reference method) × 100, while the mE rate was calculated by dividing the number of mE by the total number of strains tested × 100. Acceptable performance rates for CA should be ≥90%, whereas acceptable performance for the VME rate should be ≤3%. The ME rate should be ≤3%. For ME and mE combined, the error rate should be combined ≤7%.

## Results

### Sample description

**Fig. 2:**
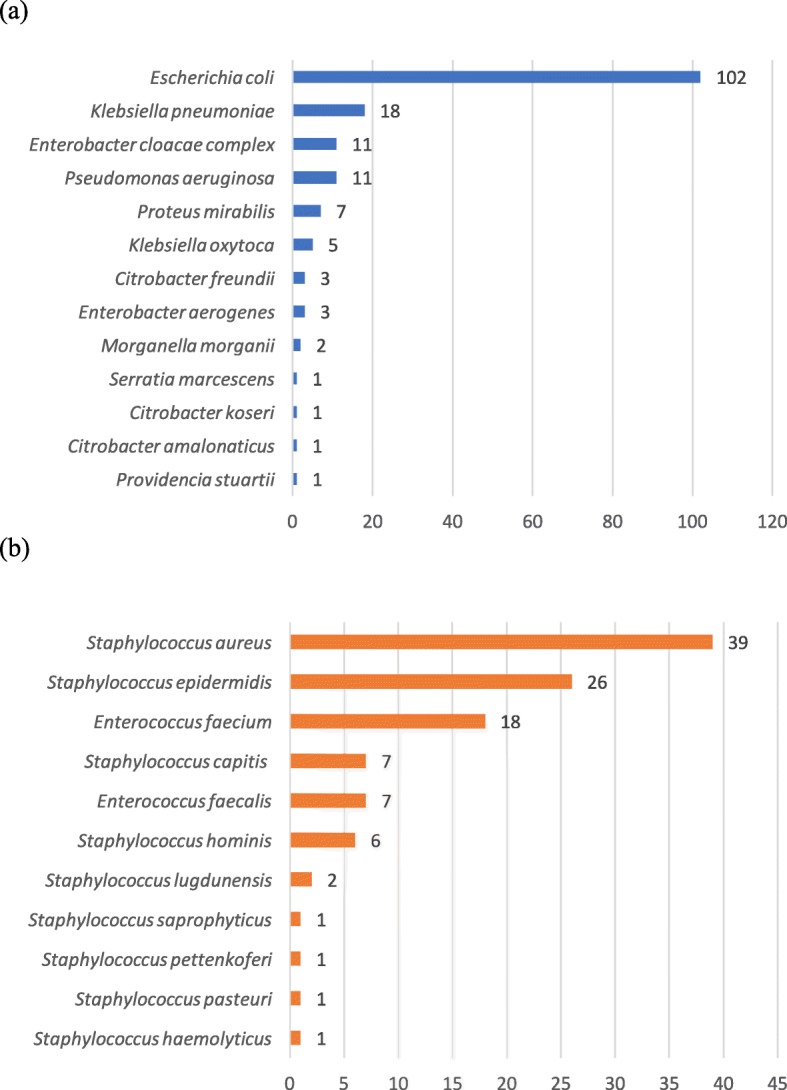
Distribution of the 275 positive blood cultures for the Gram-negative (**a**) and Gram-positive (**b**) strains

During the 2 non-consecutive 3-month periods, 288 positive blood culture bottles were included based on Gram stain results and were composed of 170 Gram-negative bacilli and 118 Gram-positive cocci. AST results of 13 (4.5%) strains were excluded from Alfred 60^AST^ performances evaluation because the antibiotic panels were not validated for their interpretation. Concerned strains were 5 streptococci (1 *Streptococcus salivarius* Group, 3 *Streptococcus mitis* Group and 1 *Streptococcus anginosus* Group), 2 *Fusobacterium periodonticum*, 1 *Micrococcus luteus*, 1 *Bacteroides fragilis*, 1 *Parvimonas micra*, 1 *Haemophilus sputorum*, 1 *Facklamia hominis* and 1 *Peptoniphilus*. Ultimately, the studied population included 166 Gram-negative bacilli and 109 Gram-positive cocci as detailed in Fig. 2a and b. With regards to the main multi-resistance profiles, 25/155 (16%) Enterobacteriaceae were ESBL-producers (17 CTX-M1, 6 CTX-M9, 1 TEM and 1 SHV), 1/11 (9%) *P. aeruginosa* expressed a VIM-carbapenemase and 3/39 (7.7%) *S. aureus* were methicillin-resistant strains. In total, 570 and 109 antibiotics were evaluated with the Alfred 60^AST^ instrument for respectively Gram-negative and Gram-positive strains.

### Direct AST for gram-negative bacilli – period I

During the first study period, AST was performed by both direct Phoenix and Alfred 60^AST^ on 70 Enterobacteriaceae and 5 *P. aeruginosa*. Results comparison led to 89.5% (248/277) CA, 8.7% (6/69) VME, 3.6% (7/197) ME and 7.5% (16/212) mE rates as detailed in Table 2. VME rates exclusively concerned cefuroxime and piperacillin-tazobactam EB respectively 19% (4/21) and 15.4% (2/13). ME rates concerned ceftazidime EB, piperacillin-tazobactam EB and ceftazidime PA (*Pseudomonas*) respectively 4.3% (2/46), 7.8% (4/51) and 20% (1/5). Ciprofloxacin showed a nearly optimal CA of 98.7% (74/75).

**Table 2:** AST results with Alfred 60^AST^ compared to direct Phoenix for Gram-negative bacteria during Period I

Antibiotic	Categorical agreement (%)	Very major errors (%)	Major errors (%)	Minor errors (%)
Cefuroxime	56/60 (93.3)	4/21 (19)	0/39	NA
Ceftazidime EB	58/68 (85.3)	0/16	2/46 (4.3)	8/68 (11.8)
Piperacillin-tazobactam EB	56/69 (81.2)	2/13 (15.4)	4/51 (7.8)	7/69 (10.1)
Ceftazidime PA	4/5 (80)	0/0	1/5 (20)	NA
Ciprofloxacin	74/75 (98.7)	0/19	0/56	1/75 (1.3)
Total	248/277 (89.5)	6/69 (8.7)	7/197 (3.6)	16/212 (7.5)

*EB* Enterobacteriaceae, *NA* not applicable (no intermediate zone), *PA Pseudomonas*

### Direct AST for gram-negative bacilli – period II

During the second study period, direct AST was performed by both direct Phoenix and Alfred 60^AST^ on 85 Enterobacteriaceae and 6 *P. aeruginosa* with a CA of 92.2% (270/293) as detailed in Table 3. No VME were observed. ME and mE rates were respectively 5.2% (13/251) and 4.3% (10/235). ME concerned cefuroxime, ceftazidime EB, piperacillin-tazobactam EB and ceftazidime PA respectively 2.2% (1/45), 12.2% (9/74), 3.6% (2/53) and 33.3% (1/3). Nearly optimal CA was observed for cefuroxime and ciprofloxacin with respectively 98.1% (51/52) and 97.7% (88/90) CA.

**Table 3:** AST results with Alfred 60^AST^ compared to direct Phoenix for Gram-negative bacteria during Period II

Antibiotic	Categorical agreement (%)	Very major errors (%)	Major errors (%)	Minor errors (%)
Cefuroxime^a^	51/52 (98.1)	0/7	1/45 (2.2)	NA
Ceftazidime EB	68/84 (81)	0/8	9/74 (12.2)	7/84 (8.3)
Piperacillin-tazobactam EB^a^	58/61 (95.1)	0/4	2/56 (3.6)	1/61 (1.6)
Ceftazidime PA	5/6 (83.3)	0/3	1/3 (33.3)	NA
Ciprofloxacin	88/90 (97.7)	0/16	0/73	2/90 (2.2)
Total	270/293 (92.2)	0/38 (0)	13/251 (5.2)	10/235 (4.3)

*EB* Enterobacteriaceae, *NA* not applicable (no intermediate zone), *PA Pseudomonas*

^a^Modified antibiotic reagent

### Direct AST for gram-positive cocci – periods I&II

Combining study periods I and II, direct AST was performed with both Alfred and Phoenix for 25 Enterococci, 45 coagulase-negative Staphylococci and 39 *S. aureus*. CA, VME, ME and mE were respectively 88.1% (96/109), 2.3% (1/44), 13.8% (9/65) and 12% (3/25) as presented in Table 4. The single VME concerned an *E. faecium* erroneously ampicilline susceptible according to Alfred 60^AST^ results. ME mainly concerned cefoxitin coagulase negative Staphylococci with 8/19 ME. Cefoxitin *Staphylococcus aureus* performed optimally with a 100% CA for all 39 tested *S.aureus* strains.

**Table 4:** AST results with Alfred 60^AST^ compared to direct Phoenix for Gram-positive bacteria during both periods

Antibiotic	Categorical agreement (%)	Very major errors (%)	Major errors (%)	Minor errors (%)
Ampicillin Enterococci	20/25 (80)	1/15 (6.7)	1/10 (10)	3/25 (12)
Cefoxitin CNS	37/45 (82.2)	0/26	8/19 (42.1)	NA
Cefoxitin SA	39/39 (100)	0/3	0/36	NA
Total	96/109 (88.1)	1/44 (2.3)	9/65 (13.8)	3/25 (12)

*CNS* coagulase-negative Staphylococci, *NA* not applicable (No intermediate zone), *SA Staphylococcus aureus*

### Turnaround time analysis

Median TAT to complete AST results with Alfred 60^AST^ was calculated at 4 h05 and 5 h55 including a sub-TAT of 1 h05 and 0 h55 to reach a 0.4–0.6 McFarland suspension for respectively Gram-positive and Gram-negative bacteria. Through the application of the workflow presented in Fig. 1 all Alfred 60^AST^ results were accessible on the same day by 5 PM.

Direct phoenix results were reached after a median TAT of 12 h34 corresponding to 12 h27 for Gram-negative bacteria and 12 h41 for Gram-positive bacteria.

## Discussion

Bacteremia is a worldwide cause of hospitalization and any kind of delay in appropriate antibiotherapy could be harmful or even fatal for the patient [16]. The wait for both identification and AST results from positive blood cultures can lead to a broad spectrum or ineffective antibiotherapy and exposes the patient to the emergence of multi-resistant bacteria [17], morbidity and mortality [18, 19]. Speeded-up positive blood culture testing is therefore an important challenge for the hospital microbiology laboratory. Many authors focused on rapid identification by MALDI-TOF MS directly on positive blood cultures [7, 20]. However, publications about direct AST methods are less prevalent and mainly address testing on AST automated systems. Beuving et al. evaluated direct inoculation of the Phoenix from positive blood cultures and showed a CA of 95.4% [21]. Similarly Pan et al. performed Vitek AST on positive blood cultures resulting into a 96.9 and 92.8% CA for respectively Gram-negative and Gram-positive bacteria [22]. At present many microbiology laboratories have introduced this direct AST approach in routine management of positive blood cultures subsequently reducing TAT to antimicrobial results with 24 h. Nonetheless results remain unavailable on the day of blood culture positivity detection. To this end, Alifax has developed an innovative AST approach based on light scattering measurements detecting the absence/presence of bacteria in a drug suspension within a few hours. In this study the Alfred 60^AST^ system and 8 selected antibiotics were challenged with 275 positive blood cultures and results were compared to those obtained with direct Phoenix testing in terms of microbiological performances and TAT. Alfred AST results for Gram-negative bacteria during period I showed moderate performances with a CA of 89.5% which is below the Cumitech acceptable performance rates [15]. Other authors reported similar to slightly higher CA results ranging between 87.7 and 97.7% [12, 23, 24]. In our study discrepancies were mainly associated with cefuroxime and piperacillin-tazobactam EB testing. Giordano et al. reported similar results for piperacillin-tazobactam EB with a CA for this antibiotic of 77.3% including 7 ME and 3 mE [24]. Conversely, this team did not observe any errors concerning cefuroxime however only 4 strains were tested. The significant amount of VME and ME led the Alifax Company to review cefuroxime and piperacillin-tazobactam EB reagents and conducted into the delivery of new antibiotic formulations. Subsequently an additional evaluation was performed on Gram-negative bacteria (Period II) with the absence of VME for both antibiotics and an improved global CA of 92.2% considered as adequate according to the Cumitech acceptable performance rates [15]. Ultimately remaining ME and mE were majorly linked to ceftazidime EB results. Our first hypothesis that errors might have been linked to the variable expression of an ESBL enzyme was countered as only 3/15 mE and 0/11 ME were associated with ESBL strains. We therefore suppose the Alfred 60^AST^ ceftazidime EB antibiotic was too weakly concentrated leading to false resistance results. Nevertheless cefotaxime is globally more sensitive for the detection of ESBL producers and should be included in the Gram-negative Alfred 60^AST^ panel when applied in routine to avoid clinical failure with third generation cephalosporins particularly for CTX-M producing Enterobacteriaceae that are cefotaxime resistant but ceftazidime susceptible. Despite only 2 ME concerning ceftazidime PA and a complete concordance concerning ciprofloxacin, AST results for *P. aeruginosa* strains are of little value as only 11 strains could be evaluated. Barnini et al. who similarly studied a population of 12 *P. aeruginosa* strains on Alfred 60^AST^ showed a total CA of 89.3% for amikacin, colistin, gentamicin, levofloxacin yet ceftazidime PA was not tested [23]. CA for AST on Gram-positive bacteria with Alfred 60^AST^ was 88.1% essentially due to a ME rate as high as 42% for cefoxitin tested on coagulase-negative Staphylococci and hereby not meeting the Cumitech acceptable performance rates [15]. Other authors obtained a Gram-positive CA between 85.1 and 93.7% [23, 24]. Similarly Barnini et al. reported cefoxitin ME rates for Staphylococci of 14.3 and 17.2% with 2 Alfred 60^AST^ test protocols [23]. Inadequately suppressing the antibiotic option of a small spectrum beta-lactam due to erroneous cefoxitin resistance detection could lead to the excess use of broad-spectrum antibiotics including vancomycin. Therefore we believe a review of the composition of the cefoxitin antibiotic for coagulase-negative Staphylococci should be considered. Finally considering the *S. aureus* population, cefoxitin showed an optimal CA as observed by others [24]. This is of importance, mainly because the rapid detection of a blood infection by a methicillin-resistant *S. aureus* requires the rapid instauration of a broad-spectrum antibiotherapy [25].

A major asset of AST testing with Alfred 60^AST^ is TAT to results calculated in our study at 4 h05 for Gram-positive bacteria and 5 h55 for Gram-negative bacteria. With this approach all positive blood cultures detected in the morning have a susceptibility profile by the end of the same day which can be considered as a drastic improvement compared with classical subculture AST testing but also compared with direct automated testing used as reference AST technique in our study. The calculated TAT of the Alfred 60^AST^ approach is quite similar to the TAT of the rapid AST technique recently introduced by EUCAST based on disk diffusion directly from positive blood cultures and interpretation of inhibition zones according to specific breakpoints after 4, 6 or 8 h. To her advantage the latter technique does not require any automated system or specific reagents however current published breakpoints are available for a limited amount of antibiotics and only 7 strains (*E. coli*, *K. pneumoniae*, *P. aeruginosa*, *E. faecalis* and *faecium*, *S. aureus* and *S. pneumoniae*). In our study the application of the EUCAST rapid AST approach would have limited the availability of AST results to 79% of the Gram-negative strains and 58.7% of the Gram-positive strains.

Our study included some drawbacks. At first our positive blood culture collection was low in multi-resistant strains and additional testing needs to be performed on methicillin-resistant *S. aureus*, ESBL and carbapenemase-producing Enterobacteriaceae as well as multi-resistant *P. aeruginosa* for a more accurate evaluation of VME rates. Alongside our evaluation of Alfred 60^AST^ was restricted to a limited panel of antibiotics chosen in accordance with our local resistance epidemiology. In a setting with high prevalence rates of multi-resistant bacteria, meropenem and vancomycin must be part of the customized antibiotic panels. Finally caution is required when global CA, VME, ME and mE are compared between publications as every team evaluates distinct antibiotic panels with different strains including varying resistance profiles.

The phase following this microbiological performances evaluation would be the introduction of Alfred 60^AST^ testing in the routine management of positive blood cultures with the assessment of the impact on patient’s outcome. A retrospective cohort study of Menon et al. concluded to a speeded-up antibiotic change in 28% of bacteremia cases through the use of susceptibility testing with disk diffusion directly from positive blood cultures versus testing on subcultured colonies [26]. In an interventional study, Verroken et al. similarly calculated a time gain of 18.2 h towards optimal antimicrobial treatment with the introduction of a speeded-up positive blood culture workflow including direct MALDI-TOF MS identification, rapid resistance detection testing and direct automated AST [27]. We believe the routine integration of Alfred 60^AST^ testing as suggested in Fig. 1 would allow analogous observations with an even shorter TAT towards antibiotic tailoring and a potential impact on patient’s mortality and length of stay. However it is important to recall that in our study Alfred 60^AST^ testing was limited to positive blood cultures detected positive until 10 AM. Extending the inclusion time frame would concurrently set back the time to available results towards evening/night hours requiring an around-the-clock running laboratory and the full-time accessibility of the clinician in charge of the concerned patient to perform instant antibiotic tailoring.

## Conclusion

Alfred 60^AST^ is an automated instrument based on light scattering technology aimed to perform antimicrobial susceptibility directly on positive blood cultures. Our evaluation shows moderate to acceptable microbiological performances and a major TAT reduction compared to current routine AST approaches. Additional optimization of certain antibiotics reagents would be an asset and improve AST results. In the near future the clinical outcome of this approach should be investigated for a rapid and effective antibiotic management of patients with bacteremia.

## Data Availability

All data generated or analyzed in this study are available upon reasonable request to the corresponding author.

## Abbreviations

AST: Antimicrobial susceptibility testing
CA: Categorical agreement percentage
CNS: Coagulase-negative Staphylococci
CUSL: Cliniques universitaires Saint-Luc
EB: Enterobacteriaceae
ESBL: Extended spectrum beta-lactamase
GNB: Gram-negative bacilli
GNC: Gram-negative cocci
GPB: Gram-positive bacilli
GPC: Gram-positive cocci
ICU: intensive care unit
MALDI-TOF MS: Matrix-assisted laser desorption ionization time-of-flight mass spectrometry
ME: Major error
mE: Minor error
NA: Not applicable (no intermediate zone)
PA: *Pseudomonas aeruginosa*
TAT: Turnaround time
VME: Very major error

## References

[1] Singer M, Deutschman CS, Seymour CW, Shankar-Hari M, Annane D, Bauer M (2016). The third international consensus definitions for sepsis and septic shock (Sepsis-3). JAMA.

[2] Shankar-Hari M, Phillips GS, Levy ML, Seymour CW, Liu VX, Deutschman CS (2016). Developing a new definition and assessing new clinical criteria for septic shock: for the third international consensus definitions for Sepsis and septic shock (Sepsis-3). JAMA.

[3] Seymour CW, Gesten F, Prescott HC, Friedrich ME, Iwashyna TJ, Phillips GS (2017). Time to treatment and mortality during mandated emergency care for Sepsis. N Engl J Med.

[4] Howell MD, Davis AM (2017). Management of Sepsis and Septic Shock. JAMA.

[5] Antibiotic resistance threats in the United States, 2013: Centers for Disease Control and Prevention, US Department of Health and Human Services; 2013. Available from: https://www.cdc.gov/drugresistance/pdf/ar-threats-2013-508.pdf

[6] Jorgensen JH, Ferraro MJ (2009). Antimicrobial susceptibility testing: a review of general principles and contemporary practices. Clin Infect Dis.

[7] Simon L, Ughetto E, Gaudart A, Degand N, Lotte R, RJJoCM R (2018). Direct identification of 80% of bacteria from blood culture bottles by MALDI-TOF MS using a 10-minute extraction protocol.

[8] Azrad M, Keness Y, Nitzan O, Pastukh N, Tkhawkho L, Freidus V (2019). Cheap and rapid in-house method for direct identification of positive blood cultures by MALDI-TOF MS technology. BMC Infect Dis.

[9] Peker N, Couto N, Sinha B, Rossen JW (2018). Diagnosis of bloodstream infections from positive blood cultures and directly from blood samples: recent developments in molecular approaches. Clin Microbiol Infect.

[10] Horing S, Massarani AS, Loffler B, Rodel J (2019). Rapid antibiotic susceptibility testing in blood culture diagnostics performed by direct inoculation using the VITEK(R)-2 and BD Phoenix platforms. Eur J Clin Microbiol Infect Dis.

[11] Charnot-Katsikas A, Tesic V, Love N, Hill B, Bethel C, Boonlayangoor S, et al. Use of the Accelerate Pheno System for Identification and Antimicrobial Susceptibility Testing of Pathogens in Positive Blood Cultures and Impact on Time to Results and Workflow. J Clin Microbiol. 2018;56(1). 10.1128/JCM.01166-17. 10.1128/JCM.01166-17 PMC5744213 29118168

[12] Sanchez-Carrillo C, Pescador P, Ricote R, Fuentes J, Losada C, Candela A, et al. Evaluation of the Alfred AST(R) system for rapid antimicrobial susceptibility testing directly from positive blood cultures. Eur J Clin Microbiol Infect Dis. 2019;38(9):1665–70. 10.1007/s10096-019-03595-y 31119576

[13] Hallin M, Denis O, Deplano A, De Mendonca R, De Ryck R, Rottiers S (2007). Genetic relatedness between methicillin-susceptible and methicillin-resistant Staphylococcus aureus: results of a national survey. J Antimicrob Chemother.

[14] Bogaerts P, Rezende de Castro R, de Mendonca R, Huang TD, Denis O, Glupczynski Y (2013). Validation of carbapenemase and extended-spectrum beta-lactamase multiplex endpoint PCR assays according to ISO 15189. J Antimicrob Chemother.

[15] Clark RB. Verification and validation of procedures in the clinical microbiology laboratory: Cumitech 31A: American society for microbiology. Washington DC: ASM Press; 2009.

[16] Kumar A, Roberts D, Wood KE, Light B, Parrillo JE, Sharma S (2006). Duration of hypotension before initiation of effective antimicrobial therapy is the critical determinant of survival in human septic shock. Crit Care Med.

[17] Armand-Lefevre L, Angebault C, Barbier F, Hamelet E, Defrance G, Ruppe E (2013). Emergence of imipenem-resistant gram-negative bacilli in intestinal flora of intensive care patients. Antimicrob Agents Chemother.

[18] Huang AM, Newton D, Kunapuli A, Gandhi TN, Washer LL, Isip J (2013). Impact of rapid organism identification via matrix-assisted laser desorption/ionization time-of-flight combined with antimicrobial stewardship team intervention in adult patients with bacteremia and candidemia. Clin Infect Dis.

[19] Perez KK, Olsen RJ, Musick WL, Cernoch PL, Davis JR, Peterson LE (2014). Integrating rapid diagnostics and antimicrobial stewardship improves outcomes in patients with antibiotic-resistant gram-negative bacteremia. J Inf Secur.

[20] Morgenthaler NG, Kostrzewa M (2015). Rapid identification of pathogens in positive blood culture of patients with sepsis: review and meta-analysis of the performance of the sepsityper kit. Int J Microbiol.

[21] Beuving J, van der Donk CF, Linssen CF, Wolffs PF, Verbon A (2011). Evaluation of direct inoculation of the BD PHOENIX system from positive BACTEC blood cultures for both gram-positive cocci and gram-negative rods. BMC Microbiol.

[22] Pan HW, Li W, Li RG, Li Y, Zhang Y, Sun EH (2018). Simple sample preparation method for direct microbial identification and susceptibility testing from positive blood cultures. Front Microbiol.

[23] Barnini S, Brucculeri V, Morici P, Ghelardi E, Florio W, Lupetti A (2016). A new rapid method for direct antimicrobial susceptibility testing of bacteria from positive blood cultures. BMC Microbiol.

[24] Giordano C, Piccoli E, Brucculeri V, Barnini S (2018). A prospective evaluation of two rapid Phenotypical antimicrobial susceptibility Technologies for the Diagnostic Stewardship of Sepsis. Biomed Res Int.

[25] Moise PA, Sakoulas G (2015). Staphylococcus aureus bacteraemia management: where do we stand and where are we going?. Evid Based Med.

[26] Menon V, Lahanas S, Janto C, Lee A (2016). Utility of direct susceptibility testing on blood cultures: is it still worthwhile?. J Med Microbiol.

[27] Verroken A, Defourny L, le Polain de Waroux O, Belkhir L, Laterre PF, Delmee M (2016). Clinical impact of MALDI-TOF MS identification and rapid susceptibility testing on adequate antimicrobial treatment in Sepsis with positive blood cultures. PLoS One.

